# The effectiveness of a de-implementation strategy to reduce low-value blood management techniques in primary hip and knee arthroplasty: a pragmatic cluster-randomized controlled trial

**DOI:** 10.1186/s13012-017-0601-0

**Published:** 2017-05-30

**Authors:** Veronique M. A. Voorn, Perla J. Marang-van de Mheen, Anja van der Hout, Stefanie N. Hofstede, Cynthia So-Osman, M. Elske van den Akker-van Marle, Ad A. Kaptein, Theo Stijnen, Ankie W. M. M. Koopman-van Gemert, Albert Dahan, Thea P. M. M. Vliet Vlieland, Rob G. H. H. Nelissen, Leti van Bodegom-Vos

**Affiliations:** 1 Department of Medical Decision Making, Leiden University Medical Center, J10-S, P.O. Box 9600, 2300 RC Leiden, The Netherlands; 2 Department of Orthopedic Surgery, Groene Hart Hospital, Bleulandweg 10, 2803 HH Gouda, The Netherlands; 3 Department of Clinical Psychology, Vrije Universiteit Amsterdam, Van der Boechorststraat 1-3, 1081 BT Amsterdam, The Netherlands; 4 Department of Transfusion Medicine, Sanquin Blood Supply, Plesmanlaan 1a, 2333 BZ Leiden, The Netherlands; 5 Department of Internal Medicine, Groene Hart Hospital, Bleulandweg 10, 2803 HH Gouda, The Netherlands; 6 Department of Medical Psychology, Leiden University Medical Center, P.O. Box 9600, 2300 RC Leiden, The Netherlands; 7 Department of Medical Statistics & Bioinformatics, Leiden University Medical Center, P.O. Box 9600, 2300 RC Leiden, The Netherlands; 8 Department of Anesthesiology, Albert Schweitzer Hospital, P.O. Box 444, 3300 AK Dordrecht, The Netherlands; 9 Department of Anesthesiology, Leiden University Medical Center, P.O. Box 9600, 2300 RC Leiden, The Netherlands; 10 Department of Orthopedics, Leiden University Medical Center, J11-R, P.O. Box 9600, 2300 RC Leiden, The Netherlands

**Keywords:** De-implementation, Low-value care, Multifaceted strategy, Hip and knee arthroplasty, Perioperative autologous blood salvage, Preoperative erythropoietin

## Abstract

**Background:**

Perioperative autologous blood salvage and preoperative erythropoietin are not (cost) effective to reduce allogeneic transfusion in primary hip and knee arthroplasty, but are still used. This study aimed to evaluate the effectiveness of a theoretically informed multifaceted strategy to de-implement these low-value blood management techniques.

**Methods:**

Twenty-one Dutch hospitals participated in this pragmatic cluster-randomized trial. At baseline, data were gathered for 924 patients from 10 intervention and 1040 patients from 11 control hospitals undergoing hip or knee arthroplasty. The intervention included a multifaceted de-implementation strategy which consisted of interactive education, feedback on blood management performance, and a comparison with benchmark hospitals, aimed at orthopedic surgeons and anesthesiologists. After the intervention, data were gathered for 997 patients from the intervention and 1096 patients from the control hospitals. The randomization outcome was revealed after the baseline measurement. Primary outcomes were use of blood salvage and erythropoietin. Secondary outcomes included postoperative hemoglobin, length of stay, allogeneic transfusions, and use of local infiltration analgesia (LIA) and tranexamic acid (TXA).

**Results:**

The use of blood salvage (OR 0.08, 95% CI 0.02 to 0.30) and erythropoietin (OR 0.30, 95% CI 0.09 to 0.97) reduced significantly over time, but did not differ between intervention and control hospitals (blood salvage OR 1.74 95% CI 0.27 to 11.39, erythropoietin OR 1.33, 95% CI 0.26 to 6.84). Postoperative hemoglobin levels were significantly higher (*β* 0.21, 95% CI 0.08 to 0.34) and length of stay shorter (*β* −0.36, 95% CI −0.64 to −0.09) in hospitals receiving the multifaceted strategy, compared with control hospitals and after adjustment for baseline. Transfusions did not differ between the intervention and control hospitals (OR 1.06, 95% CI 0.63 to 1.78). Both LIA (OR 0.0, 95% CI 0.0 to 0.0) and TXA (OR 0.3, 95% CI 0.2 to 0.5) were significantly associated with the reduction in blood salvage over time.

**Conclusions:**

Blood salvage and erythropoietin use reduced over time, but not differently between intervention and control hospitals. The reduction in blood salvage was associated with increased use of local infiltration analgesia and tranexamic acid, suggesting that de-implementation is assisted by the substitution of techniques. The reduction in blood salvage and erythropoietin did not lead to a deterioration in patient-related secondary outcomes.

**Trial registration:**

www.trialregister.nl, NTR4044

**Electronic supplementary material:**

The online version of this article (doi:10.1186/s13012-017-0601-0) contains supplementary material, which is available to authorized users.

## Background

In the last decades, abandonment of low-value care has become more important in many countries. Evidence shows that, e.g., in the USA, an estimated 30% of all medical spending is unnecessary and does not add value in care [1, 2]. Elimination or reduction of this low-value care (de-implementation) may lead to improved quality of care while reducing expenditures [3]. The importance of abandoning low-value care is underscored by the Choosing Wisely campaign which was launched in the USA in 2012 to encourage physicians and patients to engage in conversations about unnecessary tests, treatments, and procedures; the campaign is now being adopted in many other countries [1, 3]. A key element of Choosing Wisely is that medical societies create “better not to do” lists of tests, treatments, and procedures in their discipline for which there is strong evidence of overuse, potential harm, or significant and unjustifiable costs.

The next step is to translate these “better not to do” lists into action [4]. However, although there is extensive literature on how to adopt new practices (implementation) and change human behavior [5–12], the understanding of the abandonment of long-established existing techniques or practices that might have become redundant or cause overtreatment is limited [1]. It is suggested that there are fundamental differences between de-implementation and implementation, as it is harder to give up low-value care, particularly when not substituted with something else, than to adopt new and promising techniques. [13, 14] But theory or empirical evidence on how to effectively de-implement is sparse, and only limited knowledge is available about the specific agents involved in de-implementation, the relevant barriers and facilitators, and the effective interventions for successful de-implementation of low-value care [13–21].

An example of low-value care can be found in perioperative blood management. Perioperative blood loss may necessitate allogeneic red blood cell (RBC) transfusion. Therefore, to prevent allogeneic transfusions, various blood-saving techniques are used [22–26]. In total hip arthroplasty (THA) and total knee arthroplasty (TKA), blood salvage and erythropoietin (EPO) are frequently used [23, 27–29]. Blood salvage includes the collection of shed blood during and after surgery and the reinfusion of this blood intravenously. EPO is given in the preoperative stage to patients with anemia to increase the hemoglobin (Hb) level. Both techniques are used to avoid the postoperative hemoglobin level to drop below the threshold for allogeneic transfusion. The indication to use the technique is determined by an orthopedic surgeon or anesthesiologist. However, a recent meta-analysis showed that, based on RCTs published between 2010 and 2012, blood salvage did not lead to a reduction in transfused patients or in the volume of transfused blood in THA and TKA [30]. Other literature showed that EPO was effective to reduce the number of transfused patients and the volume of transfused blood [31, 32], but the costs of EPO were so high that it was considered not cost-effective in THA and TKA [29, 31–35]. Despite this evidence, both techniques are still used in clinical practice [27, 36, 37]. Additional effort is needed to reduce the use of this low-value care in patients in which the use of blood salvage and EPO is not cost-effective, taking into account the existing barriers and facilitators for de-implementation as recommended by Lorencatto et al. who calls for more theoretically informed behavior change research in transfusion [38, 39].

The aim of this study therefore was to evaluate the effectiveness of a multifaceted strategy to de-implement blood salvage and EPO in patients undergoing primary THA and TKA.

## Methods

A pragmatic cluster-randomized controlled trial was performed to assess the effectiveness of a multifaceted de-implementation strategy. The Medical Ethical Committee of the Leiden University Medical Center declared that ethical approval was not required (CME 13/132) and waived the need for written consent from patients. The trial was registered at www.trialregister.nl (ID: NTR4044) on 25 June 2013. The study protocol has been published [40].

The Dutch Orthopedic Association and the Dutch Association of Anesthesiology were involved only in the design of the intervention. They were not involved in the execution phase. There were no incentives or (financial) reimbursements either to participate in the study or to actively change during the study.

An invitation to participate was sent to all 70 Dutch hospitals and private clinics who had indicated to use either blood salvage or EPO in our preceding survey [37]. A single contact person per hospital was contacted to avoid awareness of the study goal among all participants. Exclusion criteria for both patients and hospitals are shown in Table 1. In each hospital, orthopedic surgeons were participants, except if they stated that they did not perform THA or TKA, and anesthesiologists were participants if they were involved in orthopedic blood management.

**Table 1:** Exclusion criteria for participation of hospitals and patients

Hospitals	Patients
- Hospitals considering to abandon the use of EPO or blood salvage on their own initiative - Hospitals participating in trials that interfere with the use or the discontinuation of EPO or blood salvage - Hospitals in which orthopedic surgeons or anesthesiologists are employed who are also employed at another participating hospital or hospitals with partnerships with another participating hospital - Hospitals that perform less than 50 THA or TKA within 5 months	- Bilateral surgery - Revision surgery - Patients with a malignancy (except skin cancer or cured cancers) - A serious disorder of the coronary, peripheral and/or carotid arteries, a recent myocardial infarction or CVA (past 6 months) - Untreated hypertension (diastolic BP >95 mmHg) - Patients with a pregnancy - Patients with a coagulation disorder - Patients refusing or with a contraindication for allogeneic blood transfusions - Patients with untreated anemia, Hb <10 g/dl

### Design

**Fig. 1:**
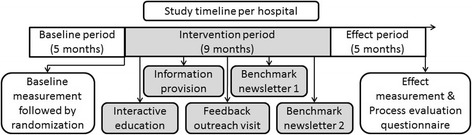
Study timeline. Components marked in *white* were applicable to all the hospitals. *Grey* components are applicable to the intervention hospitals only. Control hospitals were not contacted during the *grey* intervention period

Hospitals were randomly assigned to the intervention or control group in a 1:1 ratio using a computer-generated randomization table. Prior to randomization, hospitals were stratified by geographic location (western part versus the rest of the Netherlands) to prevent influence of regional preferences. The randomization outcome was revealed to the researchers and the hospitals’ contact person after the baseline measurement was completed. In the baseline measurement, data of individual patients, clustered within the randomized hospitals, was gathered. In Fig. 1, a timeline is shown.

### Intervention

In intervention hospitals, the participants were exposed to a multifaceted de-implementation strategy during a 9-month intervention period. The strategy was tailored to address barriers identified in our problem analysis study [38, 40]. The Theoretical Domains Framework (TDF) was used to identify relevant barriers. This framework includes 12 different domains derived from a large number of health psychology theories and their theoretical constructs [9, 41, 42]. Barriers were identified on four domains and were targeted by the four components of the de-implementation strategy (see also Additional file 1: Figure S1 for the linkage with the theoretical background):

1. Information letter/email aimed at professionals involved in blood management, other than the study participants (orthopedic surgeons and anesthesiologists), e.g., blood transfusion committee members, operating room personnel, pharmacists. A single email sent at the start of the intervention, to give an overview of literature about blood salvage and EPO use in TKA and THA, and information on the benefits of a cost-effective transfusion policy.
2. An interactive education for participants (orthopedic surgeons and anesthesiologists) with a single visit per hospital at the start of the intervention. A researcher (and also orthopedic resident) (VV) presented an overview of literature about blood salvage and EPO use in TKA and THA and information on the benefits of a cost-effective transfusion policy. Thereafter, data on the current use of blood salvage and EPO, postoperative Hb levels, transfusion rates, and length of stay (LoS) within the visited hospital were presented to specify where improvement could be achieved. During the visit, there was opportunity to openly discuss the presented information. Additionally pocket cards summarizing the literature and criteria for the use of blood salvage and EPO were handed to the participants
3. An educational outreach visit (second visit to each intervention hospital) for participants to give feedback on the use of blood salvage and EPO planned halfway the intervention period (month 5/6). The same researcher/orthopedic resident (VV) presented data on the use of blood salvage and EPO, postoperative Hb levels, transfusion rates, and length of stay (LoS) of the visited hospital from the period after the interactive education to report and promote change in transfusion policy.
4. Dissemination of two newsletters with reports on hospital performance in which own hospital data were compared to data of other intervention hospitals (anonymized) and “best practice” hospitals (two Dutch hospitals that do not use blood salvage or EPO) by email to all participants (orthopedic surgeons and anesthesiologists). The data of the “best practice” hospitals were included to emphasize the safety of not using blood salvage or EPO. The first newsletter was sent following the educational outreach visit (month 5/6), and the second newsletter was sent at least 3 months thereafter (month 8/9).

Control hospitals were not contacted during the 9-month intervention period and only received evidence by, e.g., publication of evidence in scientific journals. Likewise, there were no data gathered during the intervention period as this might create awareness and contamination of the control group. After completion of data collection for the effect measurement, the control hospitals were offered a modified intervention including the interactive education, feedback, and benchmark.

### Effect evaluation

The effect of the de-implementation strategy in intervention hospitals was compared with the usual care strategy in control hospitals. Individual patient data were gathered using a medical record review; in each hospital, two measurements took place: a baseline measurement prior to the intervention period and an effect measurement afterwards to compare the use of blood management techniques and patient outcomes between the intervention and control group, corrected for baseline. A sample size calculation was performed based on the use of blood salvage and EPO estimated in our preceding survey [37]. We assumed that if hospitals answered to frequently use these techniques, it is applied in 50% of patients. To detect a difference of 20% (from 50 to 30%) with an alpha of 0.05, a two-sided testing and power of 80%, and an intracluster correlation coefficient of 0.08, 50 patients per hospital and 20 hospitals were needed. Per measurement, a sample of 50–100 patients was retrospectively taken from the preceding 5 months by selecting the first 10–20 eligible patients of each month. Patients undergoing primary elective THA or TKA and aged ≥18 years were eligible. Exclusion criteria are shown in Table 1. With this, patients were excluded that are ineligible for elective surgery as well as patients in which blood salvage and EPO are potentially (cost)-effective.

The primary outcomes were blood salvage use (yes/no) and EPO use (yes/no). All patients were deemed eligible for blood salvage, patients with a preoperative Hb <13 g/dL were deemed eligible for EPO. Blood salvage included both intra-operative cell-saver and postoperative drainage and reinfusion; although, cell-saver was used only once. EPO-treatment was defined as a weekly dose of 600 IE/kg epoetin alfa subcutaneously starting 4 weeks before surgery, supplemented with oral iron.

Secondary outcomes evaluated patient outcomes to ensure their safety: postoperative Hb level (measured 1 day postoperatively in g/dl, continuous outcome), LoS (postoperative days, continuous outcome), and allogeneic transfusions (yes/no). The following data on patient characteristics were collected: age (years), sex (female/male), joint (hip/knee), American Society of Anesthesiologists Classification (ASA 1, 2, 3, and 4), body mass index (BMI; kg/m^2^), and preoperative Hb (g/dl).

### Process evaluation

To evaluate the de-implementation process, we gathered the following data:

1. Data about perioperative management of THA and TKA patients at patient level (alongside the data gathering for the primary and secondary outcomes at baseline and at the effect measurement) including the use of anticoagulants and antiplatelet agents, type of anesthesia (general vs. loco-regional), use of local infiltration analgesia (LIA), use of tranexamic acid (TXA), surgical approach and cementation in THA, use of tourniquet and patella prostheses in TKA, number of RBC units given, adverse events, and transfusion reactions. These data were gathered to identify changes in perioperative management of THA and TKA patients which potentially could affect the de-implementation process.
2. Information about changes in perioperative management of THA and TKA patients by field notes during all visits (including the interactive education and educational outreach visit in intervention hospitals, and data gathering in both intervention and control hospitals). During the interactive education and the educational outreach visit, a research assistant (AvdH) was present to take notes about the questions and remarks made during these meetings. During the data gathering, the person who gathered the data (the researcher VV or research assistant AvdH) made field notes about changes in perioperative management of THA and TKA patients.
3. Exposure of participants (orthopedic surgeons, anesthesiologists, and other professionals involved blood management) to the different components of the de-implementation strategy and the appreciation of the individual components by the participants [43]. For the first intervention component, the information letter aimed at professionals involved in blood management other than orthopedic surgeons and anesthesiologists, and it was reported by the research team (VV, AvdH) whether this information letter was sent and to whom. For the second (interactive education) and third (educational outreach visit) intervention component, the research assistant (avdH) that attended these meetings, reported who were attending the meetings. For the fourth component (the newsletters with reports on hospital performance/best practice), it was reported by the research team (VV, AvdH), whether and when these newsletters were sent and to whom. In addition, all participants in the intervention hospitals were asked to fill in a questionnaire (sent after completion of the intervention) including questions that evaluated the extent to which the intervention components (interactive education, educational outreach visit, and newsletters including reports on hospital performance/best practice) provided new knowledge, caused behavior change, and were appreciated on a four-point scale. For the analyses, these answer categories were dichotomized.

### Statistical analysis

#### Effect evaluation

The software package IBM SPSS 20 was used. In all analyses, a *p* value <0.05 was considered statistically significant. Differences in case mix and outcomes at baseline were compared with unpaired *t* tests for continuous variables and *χ* ^2^ tests for proportions.

To analyze the effects of the intervention on dichotomous outcomes, a generalized linear mixed model was used (i.e., logistic regression with a hospital included as a random effect) and for continuous outcomes, a linear mixed model was fitted. Both models compare the differences in outcomes in the intervention group with the control group in the effect measurement, corrected for differences at baseline and taking into account the clustering of patients within hospitals. The specified covariates included were sex, ASA classification, BMI, preoperative Hb, age, and joint. In the analyses for the primary outcomes, individual hospitals and measurement (baseline vs. effect) were added as random effects with covariance structure “unstructured.” For the secondary outcomes, individual hospitals were added as random effect with covariance structure “unstructured.” The subject-specific adjusted estimates per hospital of both measurements and effect of the intervention were presented as odds ratios (OR) for dichotomous outcomes and *β*’s for continuous outcomes.

Analyses were performed using the intention-to-treat principle. An as-treated analysis was performed in case of cross-over of hospitals. Cross-over took place if, e.g., control hospitals merged with intervention hospitals, causing participants from the control hospitals being exposed to the intervention.

#### Process evaluation

To analyze patient, the data gathered for the process evaluation were first compared between the baseline and the effect measurement using unpaired *t* tests for continuous variables and *χ* ^2^ tests for proportions. In case of significant changes between measurements, these variables were added to the previously described analyses. The qualitative data gathered for the process evaluation (field notes about changes in perioperative management of THA and TKA patients) were only used to observe trends and not formally analyzed.

The data on the exposure of participants (orthopedic surgeons, anesthesiologists, and other professionals involved blood management) to the different components of the de-implementation strategy and the appreciation of the individual components by the participants in the intervention hospitals were analyzed by using descriptive statistics.

## Results

### Study population

Twenty-one hospitals were included and randomized into 10 intervention (9 non-academic hospitals and 1 private clinic) and 11 control hospitals (all non-academic hospitals). At baseline, 924 patients were evaluated from the intervention hospitals (median 97 patients/hospital, range 75–100) and 1040 patients from the control hospitals (median 98 patients/hospital, range 64–100). In the effect measurement, data from 997 patients in the intervention hospitals were evaluated (median 100 patients/hospital, range 97–101) and 1096 patients from the control hospitals (median 100 patients/hospital, range 96–100).

At baseline, both blood salvage and EPO were more frequently used in patients from the control hospitals compared with those in the intervention hospitals (Table 2). Postoperative Hb did not differ, LoS was longer, and transfusion percentage was higher in intervention hospitals (Table 2). The distribution of patient characteristics and outcomes at the effect measurement are shown in Addtional file 1: Table S1.

**Table 2:** Patient characteristics and outcomes in intervention and control groups at baseline (unadjusted)

Characteristic	Intervention (*n* = 924)	Control (*n* = 1040)	*p* value
Joint, % knee	417 (45%)	485 (47%)	0.50
Age, years	69.3 (SD 10.0)	70.1 (SD 9.5)	0.053
Gender, % female	616 (67%)	708 (68%)	0.51
BMI, kg/m^2^	28.6 (SD 4.8)	28.7 (SD 4.9)	0.68
Smoking, %	128 (10%)	90 (13%)	*0.047*
Physical status classification^a^			*0.001*
• % ASA 1	164 (18%)	201 (20%)	
• % ASA 2	631 (71%)	653 (64%)	
• % ASA 3–4	97 (11%)	170 (17%)	
Preoperative Hb, g/dl	13.8 (SD 1.2)	13.8 (SD 1.2)	0.30
Use of LIA, %	184 (20%)	221 (21%)	0.54
Use of TXA, %	213 (24%)	190 (18%)	*0.001*
Type of anesthesia, % general anesthesia	302 (33%)	295 (28%)	*0.038*
Use of blood salvage	275 (30%)	556 (54%)	*<0.001*
Use of EPO (in EPO eligible patients)	62 (29%)	132 (51%)	*<0.001*
Postoperative Hb, g/dl	11.2 (SD 1.4)	11.2 (SD 1.4)	0.40
Length of stay	4.2 (SD 2.9)	3.8 (SD 1.8)	*<0.001*
Allogeneic transfusion, %	79 (8.5%)	62 (6.0%)	*0.027*
Number of RBC units transfused (in transfused patients)	2.5 (SD 1.44)	2.2 (SD 0.87)	0.093

^a^Due to the small number of ASA 4 patients (*n* = 1), ASA 3 and 4 are combined

### Primary outcomes

A significant reduction in blood salvage over time was found when comparing the effect measurement with the baseline of all 21 hospitals (OR 0.1, 95% CI 0.0 to 0.3). The use of blood salvage at the effect measurement, adjusted for baseline, did not differ significantly between the intervention and the control group (OR 1.7, 95% CI 0.3 to 11.4) (Table 3). A significant reduction in EPO use over time was also found (OR 0.3, 95% CI 0.1 to 1.0), again without significant differences between the intervention and control hospitals (OR 1.3, 95% CI 0.26 to 6.84), after adjustment for baseline (Table 4). The effects of the intervention varied per hospital. In intervention hospitals, the median difference between the baseline and effect measurement, based on unadjusted data, in the use of blood salvage was −11% (IQR −18 to +1%) and in the use of EPO −12% (IQR −24 to +10%). In control hospitals, the median difference, based on unadjusted data, in the use of blood salvage was −28% (IQR −45 to −3%) and in the use of EPO −17% (IQR −37 to −1%).

**Table 3:** Effects of the de-implementation strategy, measurement, and covariates on the outcome “use of blood salvage”

	OR	95% CI	*p* value
Intervention group, relative to control group	1.7	0.3 to 11.4	0.57
Time effect, effect measurement relative baseline	0.1	0.02 to 0.3	*<0.001*
Joint, knee relative to hip	4.6	3.7 to 5.7	*<0.001*
Sex, female relative to male	0.8	0.6 to 1.0	*0.042*
ASA classification, relative to 1			
• ASA 2	1.1	0.8 to 1.4	0.62
• ASA 3–4	0.8	0.5 to 1.2	0.23
BMI	1.0	1.0 to 1.0	0.27
Preoperative Hb	1.0	0.9 to 1.1	0.30
Age	1.0	1.0 to 1.0	0.58

**Table 4:** Effects of the de-implementation strategy, measurement, and covariates on the outcome “use of EPO” in EPO eligible patients (Hb <13 g/dL)

	OR	95% CI	*p* value
Intervention group, relative to control group	1.3	0.3 to 6.8	0.73
Time effect, effect measurement relative baseline	0.3	0.1 to 0.9	*0.043*
Joint, knee relative to hip	0.9	0.6 to 1.3	0.60
Sex, female relative to male	1.2	0.7 to 2.1	0.50
ASA classification, relative to 1			
• ASA 2	1.0	0.5 to 1.7	0.88
• ASA 3–4	0.5	0.3 to 1.1	0.07
BMI	1.0	1.0 to 1.0	0.42
Preoperative Hb	0.3	0.2 to 0.4	<0001
Age	1.0	1.0 to 1.0	0.97

During the study, 4 control hospitals merged with intervention hospitals and crossed over, resulting in an as-treated analysis with 14 intervention and 7 control hospitals. The as-treated analyses did not lead to new insights regarding differences between the intervention and control group (Additional file 1: Table S2a and b).

### Secondary outcomes

Postoperative Hb was significantly higher after the intervention in the intervention hospitals compared with that in controls, after adjustment for baseline (*β* 0.21, 95% CI 0.08 to 0.34). No trend over time was observed (*β* 0.02, 95% CI −0.07 to 0.11). LoS significantly reduced over time (*β* −0.40, 95% CI −0.60 to −0.02) and was, in the intervention group, significantly shorter compared with the control group, adjusted for baseline, (*β* −0.36, 95% CI −0.64 to −0.09). Allogeneic transfusions did not differ over time (OR 0.74, 95% CI 0.50 to 1.09), nor between the intervention and control hospitals (OR 1.06, 95% CI 0.63 to 1.78).

As-treated analyses on secondary outcomes showed only slight differences: Postoperative Hb was higher over time, but the significant effect of the intervention on postoperative Hb and LoS compared with those in control hospitals is no longer present (data not shown).

The reporting of adverse events and transfusion reactions was complicated by varying availability of patient records during data collections and was considered too heterogeneous to be included.

### Process evaluation

Changes were observed in perioperative management of THA and TKA patients. From the patient data, it appeared that the proportion of patients treated with LIA (a drug locally injected to reduce postoperative pain and to accelerate recovery [44–46]) increased in control hospitals from 21.3 to 40.8% and in intervention hospitals from 20.1 to 32.9%. Several physicians in the intervention hospitals mentioned during the interactive education and educational outreach visit that they hesitate to use both LIA and postoperative blood salvage to avoid drainage of the LIA and to avoid systemic effects by reinfusion of the LIA. The proportion of patients treated with TXA (a drug given to reduce perioperative blood loss [47]) increased in the control hospitals from 18.3 to 41.5% and even more in the intervention hospitals from 24.3 to 69.2%. Both techniques are frequently used as elements within multimodal rehabilitation programs [48, 49]. Eight out of ten intervention and eight out of eleven control hospitals used such a program.

Whether the observed reduction in blood salvage and EPO might be explained by the increased use of LIA and TXA was tested by adding these two variables to the previously specified models. The results are shown in Additional file 1: Table S3a and b. Both LIA (OR 0.0, 95% C 0.0 to 0.0) and TXA (OR 0.3, 95% CI 0.2 to 0.5) were significantly associated with the reduction in blood salvage over time, and adding these to the statistical models rendered the reduction of blood salvage over time as non-significant (OR 0.2, 95% CI 0.1 to 1.0). The addition of LIA and TXA to the EPO model did not change the results. Increased use of LIA and TXA was also significantly associated with all secondary outcomes (data not shown).

No other changes in perioperative management of THA and TKA patients are observed as potential explanatory factors for the effect of the de-implementation strategy.

Further, exposure of participants in the intervention hospitals to the de-implementation strategy components was assessed and the different components were evaluated. One component was not executed: “information provision by mail to other involved professionals” was deemed unnecessary by the participants. Exposure to the other components is shown in Additional file 1: Table S4. Evaluation of the executed components by participants in the intervention hospitals showed that all components had contributed to a large extent (Additional file 1: Table S5).

## Discussion

The use of blood salvage and EPO significantly reduced over time in patients undergoing THA and TKA, but similarly in the intervention and control hospitals, without an effect of the de-implementation strategy. Reduction in blood salvage was associated with increased use of LIA and TXA. A significant effect of the strategy on secondary outcomes was seen: a higher postoperative hemoglobin and a reduced length of stay in the intervention group, suggesting improved quality of care; although, the clinical relevance of these findings can be questioned.

### Findings in context of existing research

In this study, a theoretically informed de-implementation strategy to change blood management practice, tailored to previously identified barriers was tested. The rationale behind this study was that this de-implementation strategy would lead to a reduction in the use of blood salvage and EPO. We expected that the control group would continue their current practice. However, the results showed that there was a reduction in the use of blood salvage and EPO in both intervention and control groups over time. Additionally, we observed an interaction of the use of LIA and TXA with the outcomes. In a recently published review of Niven et al. [15], all studies on the de-implementation of low-value care (*n* = 38) except for one were studies without a control group. In our study, the lack of a control group would have resulted in the conclusion that use of blood salvage and EPO was reduced due to the de-implementation strategy and the observed trend in increased LIA and TXA use could have been seen as an intervention effect. This underlines the importance of including a control group in (de-)implementation studies and is thus a strength of the present study.

This study is the first study that promotes the de-implementation of blood salvage and EPO in patients undergoing THA or TKA in daily practice. It is therefore a pioneering study in a new field. Previously, studies on the implementation of transfusion guidelines, and its associated difficulties, have been published [39, 50–52]. However, this study focuses on a new phenomenon within the field of transfusion medicine, the de-implementation of low-value practices. The reduction in blood salvage over time in this study could be explained by the increased use of LIA and TXA, while the decreased use of EPO remains unclear. When considering blood salvage, this substitution of one practice by something else seemed to be an important factor. From the literature, it is known that, once established, it can be very difficult to abandon low-value clinical practices. De-implementation is not the opposite of implementation of new clinical practices and may need a different approach [13, 15]. This study is the first to suggest that substitution of low-value care may encourage de-implementation. In this study the substitutes were TXA, a simple, safe, and inexpensive blood-sparing technique [47] and LIA, a technique aimed at pain relief, which is found difficult to combine with blood salvage, as the blood salvage drain directly drains the analgesic fluid [44–46].

Although the de-implementation strategy was not effective, the result of the study is a reduction in blood salvage and EPO without deterioration of secondary outcomes related to quality of care. This substantiates that blood salvage and EPO are low-value care. Regarding blood salvage, this is in line with the literature, on which the current study is based [30, 33, 53]. Regarding the use of EPO it is striking to see that, although effective (but not cost-effective), the EPO reduction did not lead to more transfusions, lower postoperative Hb, or increased LoS. The ongoing trend that allogeneic transfusions occur less frequent in the past years, as is shown for instance in the meta-analysis of van Bodegom et al. [30], might be an explanation for this, as the benefit of EPO becomes smaller if the number of transfusions decreases.

In addition, the results of this study showed that in the intervention group the LoS of patients was significantly reduced and the postoperative Hb significantly improved as compared with that in the control hospitals. Both outcomes were used in the de-implementation strategy components to give feedback on hospitals’ performance and for benchmarking. Insight into this information may have caused awareness among participants leading to improvement on these outcomes or, for example, triggered participants to introduce LIA or TXA.

### Strengths and weaknesses of the study

A study limitation is that participants in both the intervention and control hospitals were aware of their participation in a study about blood management. We tried to avoid this by informing a single person per hospital. However, contact-persons wanted or needed to discuss participation with their colleagues. This potentially resulted in two problems: participants with intrinsic motivation to change are more willing to participate in a study that stimulates change and awareness of the study goal could be the reason that participants changed their behavior. We could not objectify this as we do not have data about non-participating hospitals.

A second limitation of the study is the data gathering. Retrospective data gathering from patient records is dependent on the availability and quality of data. The reporting of adverse events and transfusion reactions varied too much to produce reliable outcomes. On the other hand, prospective data collection would have increased the awareness among participants.

A strength of this study is that it was preceded by a problem analysis study [37, 38, 54]. In this preceding study, we identified the extent of the problem (frequency of use of blood salvage and EPO) and which barriers play a role in this specific situation of low-value care. Hence, the TDF was used to identify relevant barriers and to develop a strategy which tailored the relevant barriers.

Another strength is the addition of a process evaluation. Instead of retrospectively looking at possible explanations for results, changes in hospital policies were observed. We observed an increased use of LIA and TXA and found associations with the reduction in blood salvage. The use of LIA and TXA in a multimodal rehabilitation program may have contributed to the observed reduction in LoS (both LIA and TXA) and the increased postoperative Hb (TXA).

In addition, we could not objectify whether other factors played a role in the observed time trends in the primary outcomes and the lack of influence of the intervention, e.g., that a decrease in the waiting time for surgery makes it undesirable to treat patients with EPO or the publication of several (Dutch) studies about blood salvage convinced the control group to abandon it [33, 53, 55]. These unforeseen changes were possibly stronger facilitators than the strategy components and thereby explain the lack of effect of the intervention.

### Unanswered questions and future research

In the process evaluation, we tried to identify factors that explain the observed time effect in the reduction of blood salvage and EPO and the lack of influence of the de-implementation strategy. However, we could not objectify whether other processes played a role to the findings in this study. For instance, it is a possibility that a decrease in waiting time for surgery makes it undesirable to treat patients with EPO as this normally starts 4 weeks in advance of the surgery, or the publication of several (Dutch) studies concluding that blood salvage does not decrease allogeneic transfusion may have convinced the control group hospitals to stop the use of blood salvage [33, 53, 55]. These developments over time were possibly far stronger facilitators than any of the strategy components and thereby explain the lack of effect of the de-implementation strategy, which could not have been foreseen in the earlier problem analysis study.

Finally, a room for improvement remains, as a considerable number of patients was still treated with blood salvage and EPO. Future research should be aimed at the identification of (more) effective strategies to de-implement the use of low-value care practices to eventually improve the quality of care and lower health care costs. In this identification, a comprehensive analysis of psychological phenomena such as sticking to routines, resistance to change, peer pressure, the influence of marketing strategies of companies that supply products, and possible financial incentives should be included.

## Conclusions

This study is the first study that actively promoted to stop blood salvage and EPO in patients undergoing THA or TKA in daily practice. Although the de-implementation strategy was not effective, the result of the study is a reduction in blood salvage and EPO without deterioration of secondary outcomes related to quality of care. This substantiates that blood salvage and EPO are low-value care. Another important finding from the study is that the reduction in blood salvage was associated with the increased use of local infiltration analgesia and tranexamic acid. This suggests that that de-implementation is assisted by the substitution of techniques. Future research must reveal whether substitution is indeed an effective strategy to de-implement low-value care.

## Additional file

Additional file 1: Table S1: Patient characteristics and outcomes in the intervention and control group at the effect measurement (unadjusted). **Table S2a.** Effects of the de-implementation strategy, time, and covariates on the outcome “use of blood salvage” in an as-treated analysis. **Table S2b.** Effects of the de-implementation strategy, time, and covariates on the outcome “use of EPO” in EPO eligible patients (Hb <13 g/dL) in an as-treated analysis. **Table S3a.** Effects of the de-implementation strategy, measurement, and covariates on the outcome “use of blood salvage,” after the addition of LIA and TXA to the model. **Table S3b.** Effects of the de-implementation strategy, measurement, and covariates on the outcome “use of EPO,” after the addition of LIA and TXA to the model. **Table S4.** Exposure to de-implementation strategy components. **Table S5.** Evaluation of de-implementation strategy components. **Figure S1.** From theory to de-implementation strategy. (DOCX 46 kb)

## Abbreviations

ASA: American Society of Anesthesiologists
BMI: Body mass index
CI: Confidence interval
EPO: Erythropoietin
Hb: Hemoglobin level
IQR: Interquartile range
LIA: Local infiltration analgesia
LoS: Length of stay
OR: Odds ratio
RBC: Red blood cell
TDF: Theoretical Domains Framework
THA: Total hip arthroplasty
TKA: Total knee arthroplasty
TXA: Tranexamic acid
