# Supplementary material for: The effectiveness of a de-implementation strategy to reduce low-value blood management techniques in primary hip and knee arthroplasty: a pragmatic cluster-randomized controlled trial
Source: Implement Sci. 2017 May 30;12:72. doi: 10.1186/s13012-017-0601-0 (PMC5450044; doi:10.1186/s13012-017-0601-0)
Supplement: Additional file 1: Table S1: — Patient characteristics and outcomes in the intervention and control group at the effect measurement (unadjusted). Table S2a. Effects of the de-implementation strategy, time, and covariates on the outcome “use of blood salvage” in an as-treated analysis. Table S2b. Effects of the de-implementation strategy, time, and covariates on the outcome “use of EPO” in EPO eligible patients (Hb <13 g/dL) in an as-treated analysis. Table S3a. Effects of the de-implementation strategy, measurement, and covariates on the outcome “use of blood salvage,” after the addition of LIA and TXA to the model. Table S3b. Effects of the de-implementation strategy, measurement, and covariates on the outcome “use of EPO,” after the addition of LIA and TXA to the model. Table S4. Exposure to de-implementation strategy components. Table S5. Evaluation of de-implementation strategy components. Figure S1. From theory to de-implementation strategy. (DOCX 46 kb) [file 13012_2017_601_MOESM1_ESM.docx]

Additional file

eTable 1: Patient characteristics and outcomes in intervention and control group at the effect measurement (unadjusted)

eTable 2a: Effects of the de-implementation strategy, time and covariates on the outcome ‘use of blood salvage’ in an as-treated analysis

eTable 2b: Effects of the de-implementation strategy, time and covariates on the outcome ‘use of EPO’ in EPO eligible patients (Hb<13 g/dL) in an as-treated analysis

eTable 3a: Effects of the de-implementation strategy, measurement and covariates on the outcome ‘use of blood salvage’, after the addition of LIA and TXA to the model

eTable 3b: Effects of the de-implementation strategy, measurement and covariates on the outcome ‘use of EPO’, after the addition of LIA and TXA to the model

eTable 4: Exposure to de-implementation strategy components

eTable 5: Evaluation of de-implementation strategy components

eFigure 1: From theory to de-implementation strategy

**eTable 1: Patient characteristics and outcomes in intervention and control group at the effect measurement (unadjusted)**

| **Characteristic** | **Intervention** | **Control** |
| --- | --- | --- |
| Joint, % Knee | 465 (47%) | 549 (50%) |
| Mean age, years | 69.7 (SD 9.7) | 69.8 (SD 9.5) |
| Gender, % Female | 625 (63%) | 708 (65%) |
| Mean BMI, kg/m^2^ | 28.8 (SD 4.8) | 28.5 (SD 4.8) |
| Smoking, % | 96 (10%) | 124 (14%) |
| Physical status classification* |  |  |
| - % ASA 1 | 191 (19%) | 203 (19%) |
| - % ASA 2 | 677 (68%) | 690 (63%) |
| - % ASA 3-4 | 120 (12%) | 195 (18%) |
| Mean preoperative Hb, g/dl | 13.9 (SD 1.2) | 13.9 (SD 1.2) |
| Use of LIA, % | 328 (33%) | 446 (41%) |
| Use of TXA, % | 690 (69%) | 454 (41%) |
| Type of anaesthesia, % general anaesthesia | 237 (24%) | 287 (26%) |
| Use of blood salvage | 170 (17%) | 292 (27%) |
| Use of EPO (in EPO eligible patients) | 46 (23%) | 84 (32%) |
| Postoperative Hb | 11.5 (SD 1.4) | 11.3 (SD 1.4) |
| Length of Stay | 3.4 (SD 3.0) | 3.4 (SD 2.0) |
| Allogeneic transfusion, % | 59 (6%) | 51 (5%) |
| Number of RBC units transfused (in transfused patients) | 2.4 (SD 1.7) | 2.3 (SD 1.2) |

***** Due to the small number of ASA 4 patients (n=1), ASA 3 and 4 are combined.**eTable 2a: Effects of the de-implementation strategy, time and covariates on the outcome ‘use of blood salvage’ in an as-treated analysis**

|  | **OR** | **95% CI** | **P-Value** |
| --- | --- | --- | --- |
| Intervention group, relative to control group | 0.6 | 0.1 to 4.4 | 0.61 |
| Time effect, effect measurement relative baseline | 0.2 | 0.0 to 0.8 | **0.03** |
| Joint, knee relative to hip | 4.6 | 3.7 to 5.7 | **<0.001** |
| Sex, female relative to male | 0.8 | 0.6 to 1.0 | **0.04** |
| ASA classification relative to 1 |  |  |  |
| - ASA 2 | 1.1 | 0.8 to 1.4 | 0.68 |
| - ASA 3 | 0.8 | 0.5 to 1.2 | 0.22 |
| BMI | 1.0 | 1.0 to 1.0 | 0.27 |
| Preoperative Hb (g/dl) | 1.0 | 0.9 to 1.0 | 0.30 |
| Age | 1.0 | 1.0 to 1.0 | 0.58 |

**eTable 2b: Effects of the de-implementation strategy, time and covariates on the outcome ‘use of EPO’ in EPO eligible patients (Hb<13 g/dL) in an as-treated analysis**

|  | **OR** | **95% CI** | **P-Value** |
| --- | --- | --- | --- |
| Intervention group, relative to control group | 0.4 | 0.1 to 2.2 | 0.30 |
| Time effect, effect measurement relative baseline | 0.6 | 0.2 to 2.5 | 0.51 |
| Joint, knee relative to hip | 0.9 | 0.6 to 1.3 | 0.57 |
| Sex, female relative to male | 1.2 | 0.7 to 2.0 | 0.52 |
| ASA classification relative to 1 |  |  |  |
| - ASA 2 | 1.0 | 0.5 to 1.7 | 0.89 |
| - ASA 3 | 0.5 | 0.2 to 1.1 | 0.07 |
| BMI | 1.0 | 0.9 to 1.0 | 0.43 |
| Preoperative Hb (g/dl) | 0.3 | 0.2 to 0.4 | **<0.001** |
| Age | 1.0 | 1.0 to 1.0 | 0.95 |

**eTable 3a: Effects of the de-implementation strategy, measurement and covariates on the outcome ‘use of blood salvage’, after the addition of LIA and TXA to the model**

|  | OR | 95% CI | P-value |
| --- | --- | --- | --- |
| Intervention group, relative to control group | 1.1 | 0.1 to 8.7 | 0.9 |
| Time effect, effect measurement relative baseline | 0.2 | 0.1 to 1.0 | 0.053 |
| Local infiltration analgesia | 0.0 | 0.0 to 0.0 | **<0.001** |
| Tranexamic acid | 0.3 | 0.2 to 0.5 | **<0.001** |
| Joint, knee relative to hip | 26.0 | 18.8 to 35.9 | **<0.001** |
| Sex, female relative to male | 0.8 | 0.6 to 1.0 | **0.049** |
| ASA classification, relative to 1   - ASA 2 - ASA 3-4 | 0.9  1.2 | 0.9 to 1.7  0.6 to 1.5 | 0.27  0.69 |
| BMI | 1.0 | 1.0 to 1.0 | 0.47 |
| Preoperative Hb | 0.9 | 0.8 to 1.0 | 0.20 |
| Age | 1.0 | 1.0 to 1.0 | 0.71 |

**eTable 3b: Effects of the de-implementation strategy, measurement and covariates on the outcome ‘use of EPO’, after the addition of LIA and TXA to the model**

|  | OR | 95% CI | P-value |
| --- | --- | --- | --- |
| Intervention group, relative to control group | 1.2 | 0.3 to 6.4 | 0.80 |
| Time effect, effect measurement relative baseline | 0.3 | 0.1 to 1.0 | **0.044** |
| Local infiltration analgesia | 0.9 | 0.5 to 1.8 | 0.85 |
| Tranexamic acid | 1.2 | 0.6 to 2.4 | 0.54 |
| Joint, knee relative to hip | 0.9 | 0.6 to 1.4 | 0.66 |
| Sex, female relative to male | 1.2 | 0.7 to 2.0 | 0.56 |
| ASA classification, relative to 1   - ASA 2 - ASA 3-4 | 1.0  0.5 | 0.5 to 1.8  0.3 to 1.1 | 0.93  0.09 |
| BMI | 1.0 | 0.9 to 1.0 | 0.44 |
| Preoperative Hb | 0.3 | 0.2 to 0.4 | **<0.001** |
| Age | 1.0 | 1.0 to 1.0 | 0.93 |

**eTable 4: Exposure to de-implementation strategy components**

|  | **Interactive Education** | **Educational outreach visits** | **Reports on hospital performance/best practices** |
| --- | --- | --- | --- |
| Exposure to the component |  |  |  |
| - Orthopaedic surgeons | 27/63 attended | 14/63* attended | 63/63 received report |
| - Anaesthesiologists | 19/37 attended | 1/37** attended | 37/37 received report |

* In 3 hospitals it was unknown how many orthopaedic surgeons attended the feedback meeting
** In 1 hospital it was unknown how many anaesthesiologists attended the feedback meeting

e**Table 5: Evaluation of de-implementation strategy components (questionnaire response n=50/100)**

|  | **Interactive Education** | **Educational outreach visits** | **Reports on hospital performance/ best practices** |
| --- | --- | --- | --- |
| To what extent did individual components provide new knowledge (n=50) | | | |
| - Limited extent | 16 (32%) | 12 (24%) | 24 (48%) |
| - Great extent | 24 (48%) | 27 (54%) | 14 (28%) |
| - No opinion/component not received | 10 (20%) | 11 (22%) | 12 (24%) |
| To what extent did individual components caused behaviour change (n=50) | | | |
| - Limited extent | 21 (42%) | 24 (48%) | 24 (48%) |
| - Great extent | 20 (40%) | 16 (32%) | 14 (28%) |
| - No opinion/ component not received | 9 (18%) | 10 (20%) | 12 (24%) |
| To what extent where individual components appreciated by participants (n=50) | | | |
| - Limited extent | 13 (26%) | 10 (20%) | 12 (24%) |
| - Great extent | 26 (52%) | 28 (56%) | 25 (50%) |
| - No opinion/ component not received | 11 (22%) | 12 (24%) | 13 (26%) |

Technique

Mode

**Motivation & Goals**

- Lack of interest to save money for the society/ hospital
- Lack of benefit for delivery of care

**Beliefs about consequences**

- Pressure of suppliers to use BSMs
- Concerns about losing experience with the use of BSMs
- Concerns about the safety of patients when BSMs are stopped

**Social Influences**

- Impeded by blood management policy of other medical specialties/ blood transfusion committee
- Lack of influence of respondent on blood management policy

Information provision

Feedback

Modeling/ demonstrating of behavior by others

Interactive education

Feedback in educational outreach visit

Dissemination of reports on hospital performance/ best practices

Goal specified (desired behavior)

**Target group**: blood transfusion committee, OR personnel, pharmacists.

**Timing**: single email at start of intervention.

**By whom**: research team.

**Content**:

Overview literature about cell salvage and use of EPO in TKA en THA.

(Benefits of a) cost-effective transfusion policy.

Information letter/ email

Content

**Target group**: anesthesiologists/ orthopedic surgeons.

**Timing**: single visit at start of intervention.

**By whom**: research team.

**Duration**: 1 hour.

**Content**:

Overview literature about cell salvage and use of EPO in TKA en THA).

(Benefits of a) cost-effective transfusion policy.

Casuistry/ small assignments.

Distribution of pocket cards with cost-effective transfusion policy.

**Target group**: anesthesiologists/ orthopedic surgeons.

**Timing**: single visit half-way the intervention.

**By whom**: research team.

**Duration**: 1 hour.

**Content**:

Feedback about use of BSMs, transfusion rates, complications (transfusion/ BSM related), length of stay, costs.

Discussion about casuistry, patient safety, and limited benefits of BSMs.

**Target group**: anesthesiologists/ orthopedic surgeons.

**Timing**: 2 times during intervention period.

**By whom**: research team.

**Content**:

News mails with information on hospital performance on implementation (in comparison to other hospitals) and best practices.

**Knowledge**

- Lack of alternatives
- Lack of interest to gain additional information about stopping BSMs

Identified barriers within TDF domain

**eFigure 1: From theory to de-implementation strategy**
